# Authors’ Response to Peer Reviews of “Modeling Years of Life Lost Due to COVID-19, Socioeconomic Status, and Nonpharmaceutical Interventions: Development of a Prediction Model”

**DOI:** 10.2196/38008

**Published:** 2022-04-12

**Authors:** Jari John

**Affiliations:** 1 Institute of Political Science University of Heidelberg Heidelberg Germany

*This is the author’s response to peer-review reports for “Modeling Years of Life Lost Due to COVID-19, Socioeconomic Status, and Nonpharmaceutical Interventions: Development of a Prediction Model.”*

## Review Round 1

### Reviewer F [1]

#### General Comments

The paper [2] is very well written and is very timely. I believe that this model will be informative in that it shows how long-term solutions rather than short term are needed to avoid greater losses in the long term. However, as the authors say, the model is based on somewhat weaker empirical research, so I look forward to seeing this model validated with data.

Author response: As noted in the introduction of the paper, the data that I will explain in more detail below is largely for purposes of illustration. This is because the design of the study, for various reasons, simply does not allow for “validation with examples.” One reason is that data on the socioeconomic profile of COVID-19 deaths is not available on a global scale. The other reason is that the years of life lost (YLL) due to loss of socioeconomic status (SES) are potential YLL. Whether these YLL will materialize in the future depends on current and future policy responses to the pandemic and whether they succeed at compensating for existing and containing future socioeconomic fallout. In other words, we can only hope that the model will be refuted in 10-20 years. The paper should therefore be seen as two things: a call for action based on a plausible estimate (not an empirical assessment) and an agenda to fill an important research gap. I did, however, try to insert more examples from individual countries into the conceptual discussion to clarify the approach.

#### Specific Comments

##### Major Comments

1. I think it should be made clearer that this model is applied to US and European scenarios.

2. I believe that the paper and research are well motivated and show a necessity for more research on the proportional impact of SES on YLL.

Author response: 1. The comment is based on a misunderstanding. The paper explicitly develops a global model. It is true that the main assumptions and parameters of the model necessarily rely on findings from studies on high-income countries, simply because this is the data that is available. However, for the model parameters to better suit the context of low- and middle-income countries, various adaptations are made plausible. Additionally, the study draws on the only current existing international cross-country data set on COVID-19 deaths spanning 81 countries from all income groups (see more details below).

##### Minor Comments

3. There are several cases where the authors should correct some typos (eg, the European [the European what?] and sill vs still).

Author response: Typos were corrected.

### Anonymous [3]

This paper develops a model that compares the YLL due to COVID-19 and the potential YLL due to the socioeconomic consequences of its containment. The results highlight the importance of SES in evaluating the effect of nonpharmaceutical interventions (NPIs) during COVID-19. However, the methods, especially the empirical sample characteristics from which the life table is derived, are not clear.

#### Specific Comments

##### Major Comments

1. Needs to describe more about the data, study design, and study sample in more detail

2. Needs to discuss how the missing data was handled

3. It is important to consider a theoretical framework that can guide the selection of NPIs, indicators of SES, and the equivalent socioeconomic damages (on page 11). Right now, it is more arbitrary than scientific based.

4. The Discussion also needs to consider other factors (eg, pre-existing conditions, neighborhood resources, or occupation types). These are important social determinants of health factors.

Author response: 1. Concerning the study design [Anonymous] may have mistaken the paper for an empirical study whereas its main contribution is a conceptual innovation that is made plausible using existing data. As part of the revision, I further clarified that the data on YLL due to COVID-19 are taken from an existing study [4] and the life tables they use. Their data covers 81 countries across all income groups and is the most comprehensive global data set to date. Additional information about their sample can be obtained from the appendix of their study.

2. Missing data for low- and middle-income countries is indeed a major problem, both for the socioeconomic gap in life expectancy as well as the socioeconomic profile of COVID-19 deaths in these countries. The gap is documented in the study, and it was made plausible how assumptions from high-income country can be adapted to better suit the context of low- and middle-income countries. If there are concerns with their plausibility, I am happy to take more detailed and informative reviewer suggestions on how the assumption can be improved.

3. The comment is unclear. The paper does not aim at developing a theoretical framework for the selection of NPIs. Instead, it makes the assumption that the socioeconomic fallout in the pandemic is due to the NPIs. This is justified in the discussion section where issues of causality are taken up. The selection of SES indicators is also not arbitrary. With education and income, the two most important and widely used indicators of SES were used. It was made clear in the relevant text passages that SES corresponds to income quintiles (low 1, mid 2-4, high 5) and 3- to 4-year educational blocs following primary education (low primary, mid secondary, high tertiary). Furthermore, the paper only estimates how many more people and students would have to fall one SES group in the future due to income loss and foregone education.

4. The relevance of comorbidities is acknowledged in the COVID-19 YLL estimates but it is mainly argued that data on SES can partly proxy for comorbidities. Neighborhood resources and occupation type are indeed other important indicators of SES, but I would hope the reviewer could specify how these could be integrated in the analysis.

##### Minor Comments

1. The tables need to be adjusted in terms of the decimal points and more informative legends to guide readers.

Author response: Tables were adjusted to decimal points and more detailed descriptions of their content inserted in the main text.

## Abbreviations

NPI: nonpharmaceutical intervention
SES: socioeconomic status
YLL: years of life lost
